# Navigating the Unknown: Mental Pain, Uncertainty, and Self-Isolation in Bali and Java

**DOI:** 10.1007/s11013-025-09930-7

**Published:** 2025-07-28

**Authors:** Florin Cristea

**Affiliations:** 1 Institute of Social and Cultural Anthropology, Free University Berlin, Landoltweg 1-9, 14195 Berlin, Germany; 2 Department of Social and Cultural Anthropology, University of Münster, Studtstraße 21, 48149 Münster, Germany

**Keywords:** Severe psychiatric disorders, Indonesia, Mental pain, Uncertainty

## Abstract

Mental pain is commonly defined as an experience situated on a continuum between cognitive appraisal of the painful event and the affective disposition of the person experiencing it. Drawing on ethnographic material and interviews on severe psychiatric disorders in Bali and Java, I will try to understand what mental pain does to the person experiencing it, as well as to their immediate environment. To answer this question, I will first describe the salient attributes of mental pain as they emerged during my conversations with outpatients and observations of their milieu. These were a challenged “realness” of the experience of mental pain, its ability to take hold of one’s subjective experience, an elusive and relational quality, and a perceived ambiguous and indeterminate temporal dimension. Moreover, I will describe the uncertainties of people navigating a severe psychiatric disorder (health, sanative, social, and behavioral uncertainties), and I will suggest that the salient attributes of mental pain contribute to the makeup of these uncertainties. Finally, this article illustrates that the interrelated nature of mental pain and experienced uncertainties can inform certain illness behaviors, particularly instances of self-isolation.

## Introduction

What is mental pain? In clinical psychology, one of the most common definitions is the “perception of negative changes in the self and its functions that are accompanied by negative feelings” (Orbach et al., 2003 cited in Meerwijk & Weiss, 2014). Mental pain, therefore, appears to be situated on a continuum between cognitive attributes of the experience implied in the subjective appraisal of a painful event (physical or otherwise) and the affective disposition of the person who experiences that event. Psychologists investigate it under other terms, such as psychic pain, psychological pain, psychache (Tossani, 2013), and emotional pain (Sahi et al., 2021). Or, when triggered by negative interpersonal interactions, such as rejection or loss, it is identified as social pain (Macdonald & Leary, 2005). Neurological studies suggest that in such instances, the neural machinery involved in mental pain significantly overlaps with that of physical pain (Eisenberger et al., 2003). While the epistemological underpinnings of this finding proved to be more complex than initially interpreted, it engendered a rich body of psychological and psychiatric studies focusing on the relationship between physical pain and mental pain provoked by deleterious social experiences (Eisenberger 2012).

Anthropology does not appear to operationalize a concrete definition of mental pain. The complex relationship between emotional pain and suffering is acknowledged (e.g., Luhrmann, 2001, 275; 2007, 152; Kirmayer, 2008, 322; Throop, 2010, 7), with the latter having attracted significantly more interest in anthropological studies (Butt, 2002; Robbins, 2013). Physical pain, especially among chronic pain patients, has enjoyed some attention (Good et al., 1992a, b; Jackson 1999; Buchbinder, 2010; Throop, 2008b). In these studies, most researchers draw on the definition provided by the International Association for the Study of Pain, where pain is understood to emerge at the intersection of unpleasant emotional and sensory experiences in conjunction with actual or potential bodily damage (for the most up-to-date definition Raja et al., 2020).

What would be an appropriate anthropological method to study pain for which there is no clear physical component? According to Sara Ahmed, if pain is not simply an effect of damage to the body, “rather than considering how the feeling of pain is determined (by, for example, previous experiences), we can consider instead what the feeling of pain does” (Ahmed, 2014, 24). Several anthropologists attempted to do so by examining non-physical pain—often mentioned simply as “pain” or in conjoined form as “pain and suffering” – and how it is played out in the lives of people experiencing it.

For instance, some scholars have argued that there is creative potential to its experience (Kleinman, 2008, 15) and that one’s ability to endure and communicate pain has strong culturally determined moral underpinnings (Throop, 2008a; Jenkins & Csordas, 2020, 155; Wikan, 1990, 123). James Davies suggested that learning to navigate the intensity of pain can have transformative power if it leads to changing habitual conditions into those serving deeper needs (Davies, 2012, 42). Along similar lines, Nancy–Scheper-Hughes found that in Brazil, women who repeatedly lost their children in childbirth or due to sickness managed to overcome their pain by attributing religious meaning to their suffering (Scheper-Hughes, 1993, 529). Unni Wikan (1990, 85) described how, in Bali, pain experiences are presumed to correspond with the suffering of invisible forces and ancestors with whom one’s life is inherently intertwined.

In agreement with Sara Ahmed, in this article, I will examine what mental pain *does* by drawing on data collected during 15 months of in-depth fieldwork on the experiences of people diagnosed with severe mental illness (SMI) in Bali and Java. To answer this question, I divided the article into three sections.

First, I will approach people’s experiences of mental pain associated with a psychiatric episode (manic, depressive, psychotic) and its interpretation. Although I understand mental pain along the same lines as Orbach and his colleagues, I will further attempt to contextualize its experience by drawing from *outpatients’ own definitions* and disentangling the experience of pain from that of suffering. Where suffering is understood as a perceived threat to the integrity of the self, helplessness in the face of that threat, and exhaustion of psychosocial and personal resources for coping (Chapman & Gavrin, 1993).

The line of analysis I propose follows several authors who argued that suffering, although intimately linked to pain, is not a necessary consequence of pain (Singstock et al., 2022, 23; Kleinman, 2008, 222). This division suggests that extricating the experience of pain from that of suffering might allow for novel insights into conditions that remain obscured if we maintain our focus on the interrelated connection between them. My attention, therefore, will be on the attributes of mental pain and how they emerge and shape the lived experience of people diagnosed with a severe psychiatric disorder and their immediate environments.

Second, I theorize that the intense experience of pain – potentially perceived as “world-destroying” ((Scarry, 1985) cited in (Throop, 2010, 6)) – permeates relevant dimensions of the illness experience. However, I will focus only on one facet of the illness experience, namely on uncertainties. Uncertainties are framed as the perception of being unable to assign probabilities for outcomes that result in a discomforting or uneasy sensation, potentially affected through cognitive, emotive, or behavioral reactions, or simply by the passage of time and the perception of circumstances (Penrod, 2001, 241). Furthermore, they are about the future, about the unpredictable, odd possibilities, and irregular occurrences that can afford both positive and negative appraisal of what is unknown (good fortune/misfortune) (Boholm, 2003).

Other aspects of the illness experience (meaning-making practices, the contingencies of recovery and relapse, or illness and health-seeking behaviors) could have served just as well for the same purpose. The juxtaposition of mental pain and uncertainty merely provides a case in point. I chose uncertainty to illustrate the permeating force of mental pain because of its potential implications for illness- and health-seeking behaviors (Manderson, 2011). And although interest is increasing, the experience of uncertainty is still a domain of the health behavioral sciences that remains, to a significant extent, unexplored (Berrigan et al., 2023).

Finally, I will argue that the salient attributes of mental pain intimately contribute to the uncertainties people navigate when dealing with a severe psychiatric disorder. And that the interrelated nature of mental pain and uncertainty informs certain illness behaviors, particularly self-isolating ones.

### Research Context and Methodology

Indonesia is a socio-culturally and economically highly diverse country in Southeast Asia. After its independence in 1945, the first independent administration aimed at ideologically and methodologically disentangling from previous colonial governmentalities. Health and psychiatric care were no exceptions (Pols, 2018). For instance in Bali, at the Bangli psychiatric hospital, this translated into developing collaborations with local healers, called *balian*, who were included as regular collaborators at the hospital, as well as establishing a temple on the hospital premises that would become one of the most important temples in Bali, famous for its healing powers (Thong, 1993). In 2023, when I first visited the hospital, one psychiatrist insisted the trees on the premises represented the only remnants of their colonial past. Everything else had been torn down and rebuilt.

Until the 1990s, the psychiatric care system in Indonesia had established at least one psychiatric hospital in 26 out of its 34 provinces. It provided wide mental health coverage through community health centers [*puskesmas*] and was hailed as a model for psychiatric care for other Asian countries (Pols, 2006). However, recently, other health and economic priorities have led to significant defunding of the psychiatric care system, causing treatment gaps and human rights violations of mental health patients (Praharso et al., 2020).

My research consisted of 12 months of in-depth fieldwork from October 2023 in Bali and Java and three months of exploratory research in 2019. I interviewed and visited outpatients and their families in urban and rural areas in several districts in Bali and central Java. I conducted 33 recorded interviews with people diagnosed with a severe mental illness, sometimes one-on-one, but mostly in the presence of their families (demographic information in Table 1).

**Table 1:** Demographic Information Outpatients

	**Bali (n= 21)**	**Java (n= 12)**	**Total (n= 33)**
**Gender**			
Male	14	5	19
Female	7	7	14
**Age (range)**			
18 – 24	3	0	3
25 - 34	2	4	6
35 - 44	4	5	9
45 - 54	7	0	7
55 – 64	1	1	2
65 – 74	1	0	1
No Answer/ Refuse to Answer	3	2	5
**Education (graduated)**			
Elementary School (Sekolah Dasar)	4	0	4
Highschool (SMA / SMK/SMP)	11	4	15
Associate Degree (D1 – D3)	2	1	3
Bachelor’s Degree (S1)	1	5	6
Master’s Degree (S2)	0	1	1
No Answer / Refuse to Answer	3	1	4
**Relationship Status**			
Single	8	5	13
In a relationship/ Married	9	4	13
Separated / Divorced	1	1	2
No Answer / Refuse to Answer	3	2	5
**Income producing activities**			
Yes	10	5	15
No	8	5	13
No Answer / Refuse to Answer	3	2	5
**Income producing activities (with contract)**			
Yes	3	1	4
No	8	4	12
No Answer / Refuse to Answer	10	7	17
**Religion**			
Hindu	16	0	16
Islam	1	9	10
Christian (Catholic and Protestant)	1	3	4
Atheism	1	0	1
No Answer / Refuse to Answer	2	0	2
**Onset (Teenage or Pre-teenage)**			
Yes	14	6	20
No	7	6	13
**Urban/Rural**			
Urban	16	9	25
Rural	5	3	8

Furthermore, I collected 54 interviews with biomedical practitioners (psychiatrists, psychologists, nurses), government representatives, spiritual practitioners (*pemangku, kyai, ustaz)*, and representatives of non-governmental organizations. I collaborated with organizations that provided psychosocial reintegration programs for people diagnosed with SMI, two of which I regularly visited a few times a week. I participated in psycho-education events, psychiatric workshops, and the National Congress of Indonesian Psychiatrists in Medan, North Sumatra. Finally, I visited Hindu and Islamic centers that provided care outside biomedical institutions.

In conducting research in Bali and Java, I hoped that cultural attributes associated with Balinese-Hinduism or Javanese-Islam would emerge during data analysis, enabling a cultural comparison. Despite including similar research samples and sites, such cultural comparison never manifested due to the significant heterogeneity in illness narratives across all samples regardless of their ethnicity, religion, or locality. Consequently, I decided to engage in a locality-bound cultural perspective on SMIs and to emphasize associated differences only where they emerged and were relevant for understanding outpatients’ experiences. These instances remained marginal, but I will explore some in future sections. Moreover, biomedical psychiatry was the dominant institution in their lives, understood in a Weberian and organizational sense. Therefore, many of the differences and similarities I found were filtered through and emerged from the encounter with biomedical psychiatry and the homogenizing force of the biomedical narrative, less so from cultural dispositions related to being Balinese or Javanese.

To understand how to situate mental pain on the health-illness continuum, I asked outpatients to tell me about their experiences with non-psychiatric disease conditions and then to compare them with those of their SMIs. The Indonesian term “sakit” [*lit. pain*] was used to describe physical pain [e.g., *sakit badan*], as well as pains of the soul *[e.g., sakit jiwa*], but also to pains provoked by spirits or invisible forces. Furthermore, *sakit* could mean disease or sickness. To obtain a better notion of what kind of pain people experienced with their SMI, I first asked if outpatients remembered their last experience with pain or sickness [*sakit*], for which they visited a doctor or an alternative healer. Then, I enquired about similarities and differences to the one for which they visited a psychiatrist.

Because of the multivalent interpretations that the term *sakit* allows, in my conversation with patients and their caretakers, I focused particularly on experiences of onset and relapse that led to a hospital admission. These events coincided with a profound change in the sense of self associated with negative feelings (Cristea et al., 2024), therefore aligning with Orbach and colleagues’ framework for mental pain. In other words, in my analysis, the focus was on mental pain as an internal subjective experience (Turk & Rudy, 1992), the immediate embodied response of a psychiatric episode.

I analyzed the data collected through fieldnotes and interviews inductively (Glaser & Strauss, 1967), based on thematic analysis through repeated comparison (Braun & Clarke, 2006) using the MAXQDA data analysis tool (version 24.1.0). For the data collected through recorded interviews, I included one additional step. Following the method of recursive abstraction (Polkinghorne & Arnold, 2014), I first paraphrased the data into small summaries based on the questions from the interview guide. I repeated the process until I had reduced each interview paragraph to just a few words, which I then used for a better overview of the data. I then organized the summaries I created in this process into individual themes or merged them with the themes from the field notes.

I accessed research participants through collaborations with two local PhD candidates who supported me in data collection. All participants have given written or verbal consent to be included in this research. The research was ethically cleared by the Ethics board of the Free University Berlin, by the Indonesian National Research and Innovation Agency (BRIN), and by local government offices (Dinas Penanaman Modal) in Bali and Java.

## The Attributes of Mental Pain

### Real Pain, Fake Pain, and the Devouring Nature of Mental Pain

Answering questions about differences and similarities to other types of pain and that associated with SMIs, outpatients identified resemblance often only in general notions about reliance on medication or needing rest. The most prominent difference concerned etiology. For non-psychiatric diseases, outpatients could pinpoint the exact or at least the most probable source of their pain. Mental pain required multiple sources and stories about how the disease had unfolded. Furthermore, several outpatients considered one category physical [*jasmani/fisik/tubuh*] and the other mental [*mental/pikiran*], and hence part of two different planes of experience. Such differentiation was relatively common, especially among outpatients who exhibited what psychiatrists would call good biomedical insight, like Budi, a young shop owner and a recent father, whose account was representative of this kind of separation:

*If the problem is medical, it is true. Our body is sick […] If it is kejiwaan [can have the dual meaning of a mental problem and a problem of the soul], it is mental (sic!). Our thoughts are sick."*

It was not only outpatients that, upon recovery, challenged the “realness” of their experience. The notion that physical pains were more “real” also mirrored attitudes I encountered about psychiatric disorders among families and was a major source of tension between families, caretakers, and outpatients. Sometimes, relatives accused their family members of faking their pain to evade family responsibilities associated with Balinese or Javanese kinship responsibilities: finding a source of income, building a family, giving an heir, getting involved in ceremonies and rituals, and caring for their elders.

These morally charged interactions warrant cross-cultural reflection. Ethnographic studies on SMIs in countries of the Global North (e.g., Myers, 2015) have repeatedly highlighted that the self-hood of people diagnosed with a severe psychiatric disorder is significantly challenged by their inability to earn a living and be productive. In Bali and Java, the economical-moral markers one measured oneself by and was being measured by others were not just being self-sufficient but also having the means and aptitudes to care for others, especially close kin.

Dismissing outpatients’ narratives involved outpatients, caretakers, families, and biomedical professionals. For instance, Made, in his late 30s, acknowledged his sickness and was relatively compliant with his medication prescription. He would occasionally stop taking anti-psychotics because he wanted his body to fight the disease and the reoccurring pain on its own. When he agreed to take the medication, it was because it helped him with his dark thoughts [*pikiran berat/lit. heavy thoughts*] and suicidal ideation. However, he never abandoned his account of a large U.S.-based organization that owed him millions of dollars for services he provided telepathically, which his attending psychiatrist considered an indisputable symptom of schizophrenia, and his family often dismissed as delusions.

Such interactions epitomized a general tone of conversations that emerged not only in contexts of delusions. Especially when outpatients voiced discomfort or pain for which they had no visible evidence or mentioned anxieties and fears for which they could not provide proof, it was not uncommon for outpatients to not be taken seriously. For example, in light of his persisting narrative, other accounts of Made’s mental pain became less credible, like his sudden outbursts of deep sadness. During such occasions, he more than once shut out the family and attempted suicide. He described the thoughts he experienced during such instances as inescapable and impossible to ignore. This ability to take over one’s entire being and the consuming nature of mental pain, together with an inability to focus, were referenced by almost all outpatients. These were qualities that could override one’s experience of the immediate environment, described as an inability to take control of one’s thoughts. These experiences could then be exacerbated by the dismissal they encountered.

Returning to the comparison of experiences of mental pain to the pain that emerged from non-psychiatric diseases, several other differences proved to be informative for understanding the subjective structure of mental pain. Apart from the core difference of source and locus of pain and the associated divergence between the physical and the mental, additional pain-related dichotomies surfaced during our conversations: visible vs. invisible, physical vs. spiritual, non-hallucinatory vs. hallucinatory, possibility of recovery vs. no perspective of recovery, without effect on behavior vs. affecting behavior, control vs. lack of control, clear locus of pain vs. undetermined locus of pain, predictability vs. unpredictability.

The two opposing poles that appear to govern these dichotomies are that non-psychiatric pains were rooted in concrete, identifiable, manageable conditions. The experience of mental pain was not. It was much more ambiguous and difficult to grasp. I will further unpack some of these dichotomies as they illustrate salient attributes of mental pain and how it differentiates itself from forms of physical pain. For instance, the elusiveness of mental pain that permeated these differentiations was further complicated by an indeterminate temporal dimension that usually accompanied mental pain. Even under conditions of relative stability, no perspective of recovery and a looming fear of its return pervaded its experience.

### The Temporality of Mental Pain

For outpatients who agreed to biomedical explanations for their disease, accepting the prospect of never fully recovering and relying on medication for the rest of their lives was considered an important part of the therapeutic process. Psychiatrists and physicians argued that outpatient’s acceptance of having to comply with their medication regimens testified to their recovery and good progress. Biomedical professionals attempted to normalize the dependence on psychopharmaceuticals by comparing SMIs to diabetes or high blood pressure, hoping it would ease people’s transition from everyday personhood into their newly acquired moral careers as psychiatric outpatients.

In many cases, the opposite came about. This attempt often materialized a sense of permanence that could take a toll on people’s hopes of ever being able to build what they considered a normal life. Pak Cokot, a Hindu man in his early fifties who was diagnosed with schizophrenia and became an activist and a member of a grassroots movement for the empowerment of people with SMI, briefly illustrated the disparate temporal conditions involved with psychiatric and non-psychiatric pain:

*The similarity is that pain [sakit], must be treated to become healthy, to recover. The difference is, for a stomachache or toothache, the treatment takes a short time. In one week or two weeks, the pain [sakit] disappears. For schizophrenia, the treatment is probably for a lifetime [read: mental pain might re-emerge].*

However, even under conditions where outpatients had limited familiarity with biomedical explanatory models, the unforeseeable re-emergence of the disease and the associated pain, accompanied by distrust in the healing potential of the prescribed non-biomedical medication –called local medicine [*obat lokal*] – experientially, engendered a feeling of indeterminacy of the disease condition.

For instance, Pak Kadek and his older brother explained that Kadek’s mental pain first manifested during his tooth-filling ceremony. According to Balinese Hindu lore, he was in a state of vulnerability to supernatural forces. And someone might have used those against him through sorcery [*kena santet Bali*]. Although his family was conflicted about what might have caused the disease, they continued taking psychopharmaceuticals because they calmed Kadek down. In one of our conversations, he compared a recent experience of cough and fever with the pain for which he regularly visited the psychiatrist. The elusiveness of mental pain, as well as its ambiguous temporal dimension implied in the looming threat of relapsing, was particularly visible.

*With kejiwaan, you cannot predict when it will come. If you have heat in your body [fever], you know if it is there. If it is kejiwaan, you don’t know for sure if it is there or not. you don’t realize [sadar] you have it. You can still handle other diseases, but we are not aware of [this pain] unless there are people who make us realize we have it […]*

At the end of this brief excerpt, another characteristic of mental pain emerges. As opposed to physical conditions, mental pain was less individualized, and its experience was more socially contingent. Not only outpatients but also families and treating professionals found themselves in positions where they did not know if exhibited behaviors and health conditions were a manifestation of mental pain. In other words, the experience of mental pain was predicated on an exchange between the sufferer and their immediate environment. Given the possible serious consequences of this exchange (e.g., Biehl, 2013; Jenkins, 1991; Jenkins & Csordas, 2020; Pinto, 2014), the relationality of mental pain warrants a closer look.

#### Mental pain as a relational experience

The relationality of mental pain occurs on several levels. First, the causes of mental pain were situated in the immediate social environment. It was not uncommon for outpatients to attribute psychiatric episodes to events or conditions for which they considered themselves to be ill-equipped. Mental pain and suffering were attributed to parenting, the use of supernatural forces [*santet*], or jealousy [*cemburu*] and envy [*iri*], which had the potential to cause misfortune and sickness. Attributing mental pain to a broken heart [*sakit hati*] was relatively common. Finally, trauma was also a common explanatory model.

It is important to note that these different causes attributed to the manifestation of mental pain bear traces of outpatients’ cultural environments, including but not limited to biomedical worlds of pain and healing. *Santet, cemburu, iri, and sakit hati* point to Indonesian complex emotional regimes and invisible worlds (Good, 2004; Röttger-Rössler, 2004; Stodulka, 2016; Thajib, 2017). Parenting stereotypically implies “Asian” excessive expectations of their progeny, which was usually described by outpatients who studied past high school. Finally, trauma is characteristic not just of Indonesian psychiatric narratives but is illustrative of public and academic discourses about the colonial experience, repeated natural disasters, the complex history of violent unrest in Aceh, East Timor, Ambon, and Papua, and the controversial killings of 1965 (Spyer, 2002; Lemelson & Suryani, 2007; Good et al., 2009; Good et al., 2015; Samuels, 2019; Stodulka, 2020).

In postcolonial contexts, such as Indonesia, trauma is historically bound to interconnected forms of violence (structural, social, colonial, political, historical) (Pinto, 2020). In Indonesia and other Asian countries, European and North American notions of trauma have been mediatized through repeated international humanitarian intervention and agencies (Pols et al., 2024). Such interventions tend not just to disseminate but also normalize trauma as a concept in the contexts where these take place (Fassin and Rechtman 2009). In Indonesia, evoking trauma may also hint at traumatic political memories and hidden transcripts, faded from everyday awareness, but with a potential explosive power when revived (Good et al., 2008). For the psychiatrists and outpatients I encountered, trauma was synonymous with repeated experiences of individual violence. However, the fact that it represented a relatively common explanation for mental pain, I believe, also bears witness to a shared history of collective suffering.

Second, the experience of mental pain directly involved the immediate environment of the afflicted. It had a shared quality, situated and experienced collectively. The questioned “realness” of mental pain required the social environment to authenticate the disease occurrence. Similar to the accounts of Kadek or Budi, Padma, another participant, explained that *“[…] if it is a physical illness, you know it quickly. If it is kejiwaan, sometimes we must wait for it to worsen before we know what it is*.” For psychiatric disorders, the implications of the disease condition getting “worse” were, however, synonymous with the pain associated with a psychiatric episode and an outside evaluation of one’s experience requiring swift and sometimes violent social intervention.

Finally, the experience of mental pain was closely linked to the profound relational experience of intense emotions. It was not uncommon for outpatients to recall instances of fiercely losing control [*ngamuk*] in the presence of others. They would associate depressed and elated emotional events with particular social environments and interactions. For instance, during a relatively incoherent interview with Bu Rina, a Muslim woman in her early 40s, who occasionally sold toys on a busy tourist street in Yogyakarta, I failed to grasp what she understood to be her sickness or cause of medical discomfort. To paint a clearer picture, she sharply summarized her disease as mental emotions [*emosi mental*]*.*

*“Emosi”* held a particular weight in outpatients’ and families’ stories about instances of mental pain. I translated *emosi* as negative emotions that differentiated themselves from positive emotions [*perasaan*] in several ways. First, many outpatients located *emosi* in the head and considered them closely linked to one’s thoughts as opposed to the chest or the metaphorical heart called *hati*, the locus of *perasaan*. Second, *emosi* would emerge as part of social interactions. They were triggered by outside, usually negative events, whereas *perasaan* emerged from within the individual. *Perasaan* represented one’s emotional disposition that could arise during a social encounter but did not necessarily reflect the quality of the encounter. Third, *emosi* were associated with a lack of control and a negative impact on an individual’s behavior, as opposed to *perasaan,* which was rarely associated with either. *Perasaan* could be part of someone’s experience of a behavior*.* However, I found very few instances where *perasaan* explained why someone would behave in a certain way. *Emosi,* on the other hand, was mostly associated with (negative) conduct.

The following account of Bu Aminah is illustrative of the engrossing power of *emosi* and its relational quality and the particular place it holds in the experience of a psychiatric episode. She was a Muslim woman in her late thirties who described the onset of her disease involving a fight with her sister. Noteworthy in her story is the intensity of her emotions and being overpowered by them, but also how she repeatedly attributes pain [*sakit*] to the experience of her disease:

*It was emosi. I slammed a jar that my sister gave me. There was no place for me to vent [mencurahkan]. It was my mistake. [I was angry] and I jumped into a well. […] My emosi peaked [memuncak]. Because it hurt [Karena sakit]. Because it hurt. Because it hurt. It was only this emosi [Cuma emosi itu]. I couldn’t pour out my heart [ngak bisa mencurahkan isi hatinya]. This is this disease [sakit]*. This is what it does to you.

In summary, in this section, I described the salient attributes of mental pain by drawing on people’s reflections on their experiences with non-psychiatric diseases. The attributes that stood out were that the “realness” of mental pain was less certain than physical pain, and outpatients and caretakers could challenge its veracity. Mental pain could take hold of one’s subjective experience. It was much more elusive and difficult to grasp than physical pain and corresponded with a perceived ambiguous and indeterminate temporal plane. And it was described in more relational terms than the pain associated with non-psychiatric illness conditions. These attributes significantly impacted outpatients’ attitudes and experiences of their disease. I will illustrate this point by juxtaposing the experience of mental pain with the uncertainties that surround the experience of a severe psychiatric disorder.

## Knowing, Not Knowing, and Not Caring to Know

Psychiatric disorders as diagnosed, and mental pain as experienced, represent health conditions, respectively, conditions of illness, that are infused with uncertainty. These uncertainties emerged during illness progression and were entangled with the salient attributes of mental pain as well as with individual and social attitudes toward information-seeking. Sandra Calkins (2016) proposes a similar argument in her ethnography of navigating uncertainty in Sudan, in which she suggests that how knowledge about the unknown is engaged is crucial to differentiating between uncertainties. In Bali and Java, for instance, people may have inquired or have been informed about their disease, but they could not grasp with certainty the implications of the disease condition. They may have been looking for information they did not have and did not know how to obtain. Or may have simply avoided information related to their disease out of fear that the knowledge they would acquire could influence the outcome of the disease. Associated with these attitudes toward knowledge, I identified four types of uncertainties: health, sanative, social, and behavioral uncertainties.

### Health Uncertainties

Health uncertainties reference the disease condition or nature of the disease (both biomedical and non-biomedical explanatory models). These emerged either because of information people had, lacked, or refused to acquire. Nearly all outpatients I interacted with have had experiences with biomedical institutions and diagnostic systems, as well as non-biomedical, alternative, complementary, and/or spiritual healing traditions. Practitioners agreed with blending different treatment traditions if these practices aligned with any officially state-sanctioned religion (Islam, Hinduism, Buddhism, Protestantism, Catholicism, and Confucianism).

Outpatients did not experience combining different treatment traditions as a contradiction. First, these were perceived to be two faces of the same coin or a continuation of each other. They provided the answers to two different sets of questions. Biomedical psychiatry answered the question of **what** caused their mental pain and subsequent suffering. The answer usually entailed the brain, genes, or a perceived stressful environment. Alternative and complementary treatment traditions were more concerned with **who** was responsible for their pain. And the answer was socially and culturally more complex and dynamic. Sources of mental pain could have stemmed from ancestors, invisible worlds of spirits and unseeable forces, fractured social relationships, strained relationships with kin, and unfulfilled or unrequited love.

Second, some outpatients reported good experiences after following the advice of non-biomedical healers about food consumption and mending or breaking certain social relationships. They perceived that the advice given by alternative healers added value to their treatment. Comparatively, in psychiatric encounters, they considered that psychiatrists and physicians shared little information. Regardless, health uncertainties would emerge for both biomedical and non-biomedical explanatory models.

In terms of biomedical explanations, I conducted hours-long interviews about the diseases called “schizophrenia” and “depression” caused by, among other things, burdens [*beban*]. Only to be candidly asked “What is schizophrenia?” or “What is depression?,” when I enquired if there was any information they thought they were missing about their disease. Many explained that they received little explanation when they went to the hospital, other than that they needed medication, possibly for the rest of their life. They had some notion about heredity (understood as transmitted by blood, not a debt of or to the ancestors), but they could not comprehend why, then, they alone in their family would be sick. In other words, outpatients and caretakers appeared to hold biomedical knowledge of the disease, which they acquired by themselves or in conversations with their attending physician but remained unsure of what this information meant for them, their environment, and their future.

Furthermore, confusion did not only ensue from the experience with biomedical institutions and labels. They could also emerge from interactions with alternative and complementary healing practices. After years of attending *ruqiah* [*Islamic healing method based on Quran and Ayat*], organizing ceremonies, attending *melukat [Balinese Hindu water healing and purification ritual]* sessions, and visiting priests and other non-biomedical healers, many families confessed that they still did not know what or who was to blame for their pain and misfortune. The pervading and persistent nature of SMIs placed the experience of mental pain and associated illness outside of common references of healing, which, in other circumstances, could be dealt with and resolved with available resources. The ambivalence of the experience of mental pain and being unsure about the realness of its experience, especially during repeated instances of relapse, only added to the confusion.

In fact, the argument can be made that these attributes of mental pain were not only intimately linked to health uncertainties but that they were constitutive elements of these uncertainties. Diseases of the body and associated pains – perceived as more real – did not warrant extensive questioning of the source or nature of the disease. As long as treatments worked, whether biomedical or non-biomedical, further investigations were unnecessary. The ambiguity of mental pain could lead families and outpatients to question the nature of the disease and their own experiences.

### Sanative Uncertainties

Sanative uncertainties related to treatment, coping, and recovery. These occurred when people had some notion about their disease or what might have triggered their disease condition (biomedical and non-biomedical explanatory models). Many outpatients and families, according to practitioners, had good insight into their disease condition and were relatively medication-compliant. However, good insight also meant high expectations from biomedical interventions, which would seldom be achieved.

Many outpatients had a history of repeated hospitalization, and the disappointment of never completely recovering bound together many of the narratives I collected and affected patients’ and families’ attitudes toward the past and the future. The past was cobbled in illness histories of roads not taken and chances missed. Questions about the prospect of new hospitalizations, dependency on medication, and fear of the side effects of long-term medication intake clouded the future. These questions usually remained unaddressed in hospitals and clinics.

In contrast, the experience with alternative and complementary healers was much more generous in terms of explanations and answers (see also (Lemelson, 2004)). Although treatments provided by non-biomedical healers were much more diverse than the ones acquired in hospitals and clinics and varied greatly from one healer to the next, there were similarities in terms of form, if not in content. First, these encounters were more confident. Causes were identified, and solutions were proposed. A recovery plan was laid out. Second, families would be much more involved in treatment. There were many instances when healers requested the patient to be left alone with the patient, sometimes even for weeks. However, according to families, they were still involved in collecting ingredients for medication, or in organizing ceremonies or rituals, as well as participating in collective healing practices. Third, the healing powers of the treatment were not associated with elements considered harmful in the distant future, like the chemicals of psychopharmaceuticals. At the same time, similar to biomedical treatments, although patients might have experienced some relief, the results were not lasting.

Given that neither biomedical nor non-biomedical treatments could promise a full recovery, several outpatients confided that they considered that all treatments had an uncertain outcome. They came with different results. None guaranteed a full recovery. Therefore, an inability to predict the outcome left plenty of room for experimentation. In other words, sanative uncertainties were bound to an impending sense that mental pain could reappear and that no matter how hard families and outpatients tried, there was no real perspective of recovery. Sanative uncertainties, for this reason, were interrelated with the indeterminate temporal dimension of mental pain.

### Social Uncertainties

Social uncertainties emerged in the context of unfulfilled desires, improving one’s social life, fulfilling kinship duties and communal expectations, or improving financial situations. Many patients did not have any income-producing activities. Those who worked often had menial jobs such as sorting garbage at the materials recovery facility or directing motorcycles and cars into parking lots. The associated uncertainties appeared to be independent of the knowledge they had acquired. Even under conditions of not wanting to know about their illness, many outpatients were aware of their social isolation and limitations.

In my work, I encountered a shared concern about outpatients’ futures. What would happen once their family could no longer care for them? Would they ever be able to meet someone who accepted their health condition? Would they ever be able to provide for themselves? Such questions negatively influenced the hope of ever recovering. Outpatients anticipated that avoiding pain and achieving positive health could occur in conjunction with an improved social standing – like finding a job or getting married – from which they were excluded due to their illness condition. A vicious cycle that was not lost on them and that many perceived as very difficult to break.

In Bali particularly, families were unsure if they could involve their sick relative in ceremonies. Sometimes, the outpatients themselves refused to participate. Other times, the family objected to involving the patient out of fear that the emotionally charged environments of ceremonies and processions might lead to another psychiatric episode. Families would, therefore, have to cover the financial and organizational costs of these ceremonies without the contribution of outpatients. These responsibilities were supposed to be shared. Men had to pay for the cost of the ceremonies while women were in charge of preparing the event. Outpatients excused themselves or were excused from these responsibilities. Not participating in these important aspects of social life contributed to a decreased sense of personhood among patients and concerns about them ever achieving being fully “Balinese.”

The relational character of mental pain contributed to the structure of social uncertainties in subtle ways. Outpatients may have avoided social interactions because they associated mental pain with certain social actions and actors. Furthermore, research suggests that outpatients’ recovery and reintegration in the community were significantly hindered by their inability to reach emotional attunement to their social environment and violations of the Balinese and Javanese precept of harmonious living together [*rukun*] (Subandi, 2015; Subandi and Good 2018, Suryani, Lesmana, and Tiliopoulos 2011). This inability may be linked to mental pain manifesting in conjunction with intense emotions that engender the intersubjective nature of the experience of mental pain. *Emosi*, therefore, impinged not only on individual experiences. They affected a shared social life that valued harmony, brought about by patients not knowing how to navigate their emotional and social environments.

### Behavioral Uncertainties

Behavioral uncertainties refer to an inability to adapt, understand, or predict one’s own or other people’s behaviors. Similar to social uncertainties, these emerged irrespective of held knowledge about the disease condition and were rather experiential.^1^

Several outpatients felt unsure of how to behave so as not to be perceived as mad, didn’t know how their immediate environment would act in their presence, struggled to comprehend their actions in the past, and feared their actions in the future. These were distinct from social uncertainties in that they were sometimes related to but separate from social expectations. Nevertheless, these uncertainties illuminated the tense relationship of outpatients with families and social peers, visible in instances when outpatients would choose to self-isolate so their families would not misinterpret their actions and have them admitted to the hospital.

Moreover, behavioral uncertainties associated with the initial experience of mental pain and the illness progression challenged the pre-illness personhood of afflicted individuals. Mas Sarip, for instance, a young Muslim male in his late 20s from a village not far from Yogyakarta, reflected on how the unexpected behaviors he associated with the onset of the disease intruded on his self-image. His experience and unanswered questions illustrated a common concern about perceived changes in subjectivity and suggested that the uncertainties people experience cascade from one another.

*What is wrong with me? How come I am like this? How come the disease is like this? If I take the medicine regularly, will it be different? […] I would not have thought this of myself. I wouldn’t have thought that I would do something like this [giving away things from his parents’ home]. That there would be an impulse [dorongan] like that.*

In the case of behavioral uncertainties, the relational character of mental pain and its capacity to take hold of one’s subjective experience are particularly visible. Concerning its relationality, this type of uncertainty developed because of the shared emotional tension associated with onset or relapse. However, the attribute that dominated behavioral uncertainties was the ability of mental pain to capture one’s subjective experience. It was because of this ability that individual behaviors became unpredictable and others’ behaviors incomprehensible.

## Uncertainties, Mental Pain, and Self-Isolation

In sum, the salient attributes of mental pain were a challenged “realness” or veracity of the experience of mental pain; mental pain could take hold of and consume one’s subjective experience; it was much more elusive and difficult to grasp than physical pain; it corresponded to a perceived ambiguous and indeterminate temporal plane; finally, people described the experience of mental pain in relational terms.

**Fig. 1:**
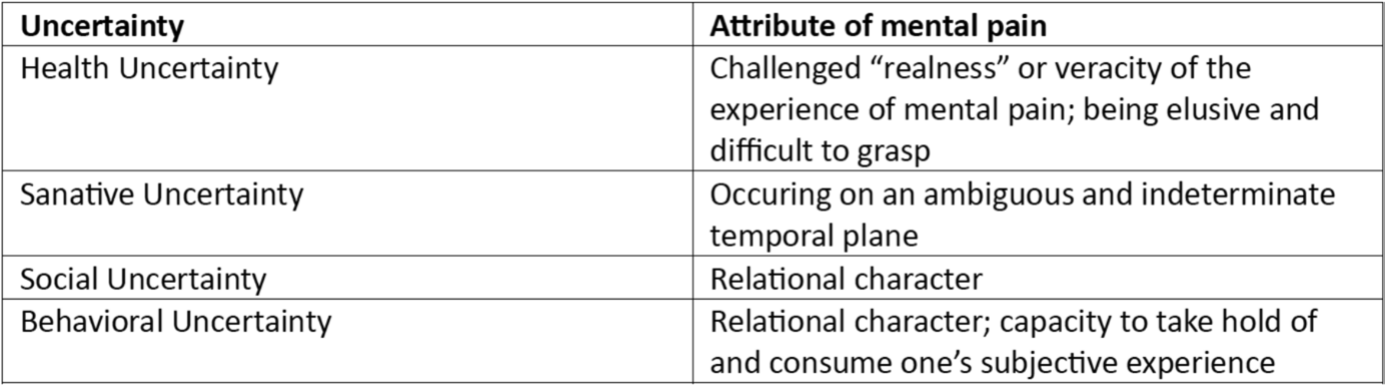
Relationship between experienced uncertainties and the attributes of mental pain

Furthermore, illness-related uncertainties were intertwined with these attributes. Health uncertainties were linked with the challenged realness and elusive quality of the experienced pain. Sanative uncertainties consisted of an ambiguous time dimension. Both social and behavioral uncertainties mirrored the relationality of mental pain. Behavioral uncertainties emerged out of mental pain’s ability to take over one’s subjective experience (Figure 1).

In this section, the argument I propose is that the intimate relationship between experienced uncertainties and the main attributes of mental pain had consequences for avoidant behaviors. Considering that a vast body of literature is dedicated to determinants of isolating people diagnosed with severe psychiatric disorders and their families (e.g., Corrigan and Miller 2004), my focus will be only on outpatients’ instances of self-isolation.

Deciding to self-isolate could result from the indeterminate temporal dimension of mental pain associated with sanative uncertainties, in addition to an inability to grasp what or who was making them sick (health uncertainties). If the cause was situated in the social environment, the re-occurrence of mental pain confirmed that someone or something continued making outpatients sick, leading to distrust and tensions between patients, their families, and their caretakers.

Furthermore, the intense experience of mental pain and the fear and unpredictability of relapsing, together with an inability to predict one’s own and others’ behaviors, could cause outpatients to stop engaging with their social world. The social, economic, and cultural barriers to an active life described earlier – like not involving outpatients in ceremonies or social activities that required financial contributions – were, therefore, exacerbated by the perceived threat of re-emerging pain. Behavioral uncertainties were a contributing factor because of patients’ fear that their mental pain could override their understanding of the environment and their actions.

Additionally, the relational character of mental pain and corresponding social and behavioral uncertainties contributed to a sense of estrangement. Self-isolation could derive from avoiding the intense negative emotions and pain associated with certain social interactions. No firm perspective for the future would create social tensions because of not living up to one’s family’s expectations. The worry of one’s social exclusion further complicated these relationships. Several outpatients explained that since the onset of their disease, even the slightest blunder, like unexpectedly singing, would be regarded as a sign of relapse. They, therefore, self-isolated or even refused to talk because they hoped that as long as they didn’t behave or talk strangely, people would not suspect they were sick or relapsing.

## Instead of a Conclusion. Possibilities of an Anthropological Perspective of Mental Pain

I began this article by suggesting that a concrete conceptualization of mental pain is virtually missing in anthropology. While anthropologists could learn from existing debates in psychology and psychiatry, they could also contribute to these discussions by situating mental pain in its social, cultural, political, and economic context. To do so, I emphasized the salient attributes of mental pain and how they play out in the social realities of people diagnosed with severe psychiatric disorders in Bali and Java.

One attribute was a challenged realness of mental pain. This argument aligns with anthropological studies on chronic physical pain that highlight how the familial environment challenges the experiences of afflicted individuals (Kleinman et al., 1992, 11; Jackson, 1992, 140). That the experience of mental pain could be contested by outpatients themselves, however, represents a particularity of my findings. Furthermore, mental pain could override one’s subjective experience of the immediate environment. It proved much more elusive than physical pains and was situated within an ambiguous and indeterminate temporal dimension. Finally, mental pain was experienced and described in relational terms.

It is important to note that the relationality of mental pain occurs in terms of explanatory models but also as a shared experience. For example, I argued that repeatedly invoking traumatic experiences as a source for mental pain may hint toward a shared history of collective suffering. In addition, psychiatric episodes and the associated mental pain involved more than one participant, foremost because of the emotional charge of these events. In other words, instances of mental pain had an emotive quality (Stodulka, 2016, 31-32). They were contagious and aimed to elicit particular emotions in those who participated in or witnessed such episodes. And the usually negative emotional charge of these interactions significantly hindered patients’ and their families’ attempts to participate in community life.

Studies in Indonesia, particularly from Java and Bali, suggest that the distinction between self and others is blurred, and personhood emerges in interaction with others (Good, 2004). This relational quality of personhood depends on adhering to strict emotional self-regulation and avoiding displays of intense negative emotions within an affective context that highly values emotional composure (Geertz, 1973; Wikan, 1990; Stodulka, 2016, Subandi & Good 2018). Not being versed in navigating this complex emotional landscape may lead to people not being recognized as fully Javanese (Beatty 2019) or Balinese (Jennaway, 2002).

Furthermore, I assumed that certain dimensions of the illness experience must be infused with characteristics of mental pain because of the profound rupture it caused in one’s perception of self and others. Namely, I suggested that the uncertainties people navigated as part of their illness experience – health, sanative, social, and behavioral uncertainties – were permeated and shaped by these different attributes of mental pain. Despite anthropological evidence to the contrary (Good, 1994, 153; Whyte, 1997, 216; Dilger, 2005, 204 ff), the prevailing stance in the social sciences investigating uncertainty appears to be the uncertainty reduction or increasing predictability theory developed by Berger and Calabrese (1975), which suggests that people have a natural drive toward reducing uncertainty (for more recent examples, see (Pavlou et al., 2007; Grundmann et al., 2021)). I argued that how people deal with and experience uncertainty is less part of some innate drive but rather entangled with the salient attributes of mental pain and people’s attitudes toward knowledge and information-seeking.

For instance, Health Uncertainties, about the disease condition or nature of the disease, emerged in a context where some participants refused to acquire knowledge about their disease out of fear that it might influence its outcome. Therefore, maintaining these uncertainties could correspond with individual coping strategies and maintaining possibilities for the future (Krause, 2006). I suggested that the ambivalence and questioning of the realness of mental pain enable such positive attitudes toward uncertainty.

Similarly, Sanative Uncertainties, about treatment, coping, and recovery, were tied to and interrelated with the indeterminate temporal dimension of mental pain. Social uncertainties emerged in the context of unfulfilled desires, and not knowing how to improve one’s social life, fulfilling kinship duties and communal expectations, or improving one’s financial situation. These were intimately connected to the relational character of mental pain, in particular because of its emotive quality. Finally, Behavioral Uncertainties referred to an inability to adapt, understand, or predict one’s own or other people’s behaviors, and were, therefore, bound to the relational character of mental pain and dominantly the ability of mental pain to capture one’s subjective experience.

Furthermore, I argued that turning our attention to mental pain can illuminate aspects of the illness experience that remain obscured if researchers only pay attention to the intertwined relationship of pain and suffering. By juxtaposing mental pain and uncertainty, I suggested not only that the salient attributes of mental pain contribute to the makeup of uncertainty but that because of their complementary relationship, we gain significant insight into the motivations for self-isolating behaviors (e.g., in anticipation of the re-occurrence of mental pain and being unable to anticipate one’s own and other people’s behaviors). While this argument in no way undermines the social and cultural determinants of marginalization, it emphasizes the role that outpatients might play in perpetuating instances of isolation.

Already in the 70s, Sue Estroff (1981) conveyed in her work on psychiatric outpatient communities in the U.S. the need to investigate the role of “clients” in exclusionary practices during the process of attempted reintegration. Understanding this aspect of the illness behavior, she argued, was virtually missing, with a clear research preference for studying communities and their marginalizing attitudes and behaviors. A few exceptions notwithstanding (e.g., Jenkins & Csordas, 2020; Littlewood, 2002), with a focus on marginalized communities in Java (Stodulka, 2016)), judging by the priorities set by the Lancet Commission on ending stigma and discrimination in mental health (Thornicroft et al., 2022), there still appears to be relatively little interest in patients’ and outpatients’ agency and their involvement in circumstances of marginalization. The nexus of social and behavioral uncertainties and the corresponding attributes of mental pain, particularly its relational quality, could pave the way for future investigations of marginalization that not only focus on the community or the affected individual but on the intersubjective nature of their encounter.

Finally, there are several parallels between the findings I presented and anthropological studies of chronic physical pain: both physical and mental pain debilitate and unmake one’s sense of self into the world (Good, 1992, 40; Kleinman, 2008, 136; Throop, 2010, 276); both are ambiguous (Honkasalo, 2001); the social and medical environment of the sufferer may question the authenticity of the pain experience (Jackson, 1992, 147); biomedical and non-biomedical treatment provide little resolution (Kleinman et al., 1992, 6); they are intersubjective (idem 9); and have a particular time dimension (Good, 1994, 126).

Considering these similarities, I believe some of the recommendations for future research that DelVecchio Good and her colleagues (1992a, b) laid out in their work on chronic pain apply just as well to the study of mental pain. For instance, systematic comparative studies of the macro-social processes involved in the workings of local uncertainty and mental pain structures could provide valuable insights into the cultural composition of both categories. This could illuminate culturally sensitive pathways to understanding, navigating, and dealing with mental pain and uncertainty.

In addition, investigations of the relationship between the experience of mental pain and narratives of sufferers and biomedical and non-biomedical professionals could provide valuable insight into the phenomenology of mental pain and how it plays out in local worlds. Furthermore, it would be interesting to examine if available cultural scripts for dealing with physical pain inform attitudes and knowledge about mental pain. However, to enable such research, systematic cross-cultural studies on the structure and attributes of mental pain would be required to develop anthropologically coherent tools for the comparative research of mental pain. I hope my article can provide a first step in this direction.

## Data Availability

Due to the qualitative nature of my study, making the qualitative data I collected freely available may pose a risk to the participant’s anonymity. For this reason, I cannot share recorded data. The demographic information of all participants is available in Figure 1 of the manuscript.

## Footnotes

1 In Feldman Hall and Shenhav (2019) and Brashers and colleagues (2003), these would be closer to social uncertainties
